# An unyielding challenge for refractory hyponatremia in neuroendocrine cervix carcinoma: a case report

**DOI:** 10.3389/fendo.2025.1451157

**Published:** 2025-02-28

**Authors:** Minzhen Li, Rutie Yin, Lan Zhong, Liang Song

**Affiliations:** ^1^ Department of Gynecology and Obstetrics, West China Second University Hospital, Sichuan University, Chengdu, China; ^2^ Key Laboratory of Birth Defects and Related Diseases of Women and Children (Sichuan University), Ministry of Education, Chengdu, China

**Keywords:** neuroendocrine neoplasm, NECC, severe hyponatremia, electrolyte imbalance, next-generation sequencing

## Abstract

Neuroendocrine neoplasms (NENs) originate from peptidergic neurons and neuroendocrine cells, possessing endocrine functions, and are commonly found in the gastrointestinal tract, pancreas, and lungs. Neuroendocrine cervix carcinoma (NECC) is infrequent, prone to early dissemination and distant metastasis, and generally has a poor prognosis. The presence of electrolyte imbalance in such cases is even rarer. Here, we present a case of advanced NECC patient who developed refractory hyponatremia, accompanied by severe clinical symptoms such as palpitations, chest tightness, hematemesis, and delirium. Despite extensive investigations, all efforts to elucidate the underlying causes of hyponatremia were negative, suggesting a multifactorial etiology. Next-generation sequencing was also employed to explore the underlying mechanisms at the genetic level. Managing this case posed significant challenges for gynecologic oncologists, as the patient showed minimal response to various treatments, including sodium supplementation, fluid restriction or replenishment, chemotherapy modification, and the use of vasopressin-2 receptor antagonist. This case underscores the importance of monitoring and managing electrolytes in patients with gynecologic NENs, even though the exact mechanisms of such imbalances may remain elusive.

## 1 Introduction

Neuroendocrine neoplasms (NENs) are a heterogeneous group of tumors originating from peptidergic neurons and neuroendocrine cells, capable of occurring in various tissues, predominantly in the gastrointestinal tract, pancreas, and lungs. In female reproductive organs, NENs are rare, with the cervix being the most common site, followed by the corpus uteri and the ovaries/fallopian tubes. Neuroendocrine cervix carcinoma (NECC) accounts for only 1% to 1.5% of all cervical tumors (1), and there are an estimated 5,000 to 11,000 new cases of NECC worldwide each year. In 2014, the World Health Organization (WHO) classified NECC into four categories: high-grade (small cell NECC and large cell NECC) and low-grade (carcinoid and atypical carcinoid) NENs (2). Compared to squamous cell carcinoma and adenocarcinoma, NECC tends to exhibit early local spread and distant metastasis, characterized by a higher degree of malignancy and poorer prognosis (3). The 5-year overall survival (OS) rate is 30-60% for patients with early-stage disease and 0-15% for those with advanced metastatic disease (4).

Electrolyte imbalances are relatively common in cancer, particularly in NENs with ectopic endocrine functions. A serum sodium concentration below 135 mmol/L is considered hyponatremia. Mild hyponatremia (serum sodium levels ranging from 130 to 135 mmol/L) can result in delays in therapy schedules, prolonged hospital stays, and a deterioration in the patient’s overall quality of life and general condition. More pronounced hyponatremia (serum sodium levels below 125 mmol/L) can lead to neurological and psychiatric symptoms such as headaches, vomiting, fatigue, muscle weakness or cramps, and even seizures. Severe hyponatremia was reported in about 20% of patients with small cell lung cancer (SCLC) (5) and may be associated with various factors, including paraneoplastic syndrome caused by the tumor itself (such as syndrome of inappropriate antidiuretic hormone secretion (SIADH)), side effects of antitumor therapy, comorbidities, and the use of specific medications (5). However, cases of severe hyponatremia in NECC were rarely reported and all associated with SIADH. After surgical resection and chemoradiotherapy, hyponatremia was well controlled in all cases (6–9).

In this case, we discuss a patient with advanced NECC who developed persistent severe hyponatremia of unknown origin, accompanied by grievous clinical symptoms such as chest tightness, hematemesis, and delirium. Despite changing chemotherapy drugs, controlling fluid intake or rehydrating, and administering a specific vasopressin-2 receptor (V2R) antagonist (treatment for SIADH), the patient’s condition did not improve, necessitating long-term sodium supplementation. Next-generation sequencing (NGS) of NECC component was conducted, and a comprehensive literature review was undertaken to elucidate the potential etiology. We aim to highlight the importance of electrolyte monitoring and management in patients with gynecological NENs for clinical practitioners.

## 2 Case presentation

**Figure 1:**
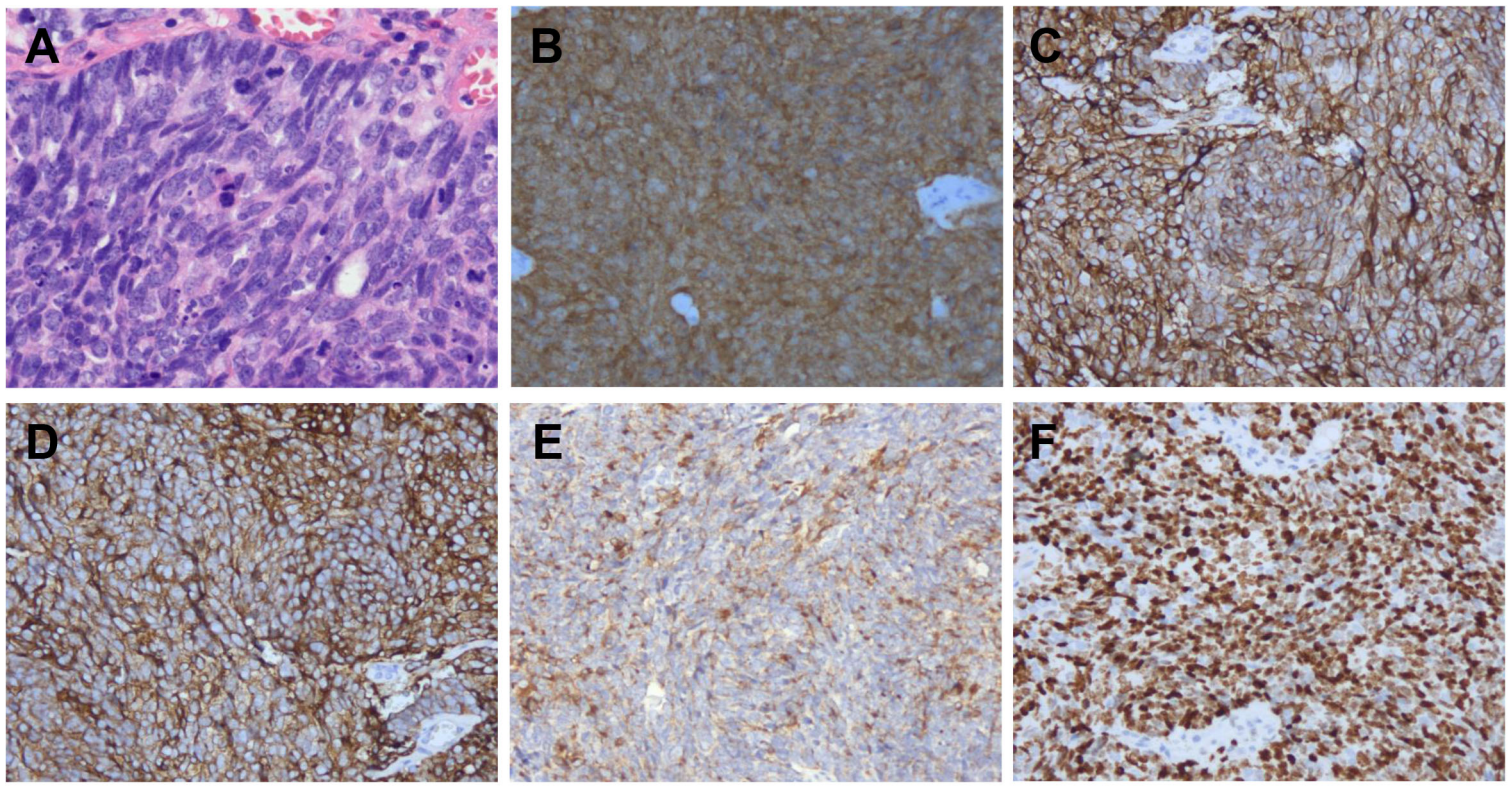
Pathological findings. Hematoxylin and eosin (H&E) staining **(A)** was performed, and p16 **(B)**, CD56 **(C)**, Syn **(D)**, CgA **(E)**, and Ki67 **(F)** were evaluated by immunohistochemistry in tumor lesion (200×).

**Figure 2:**
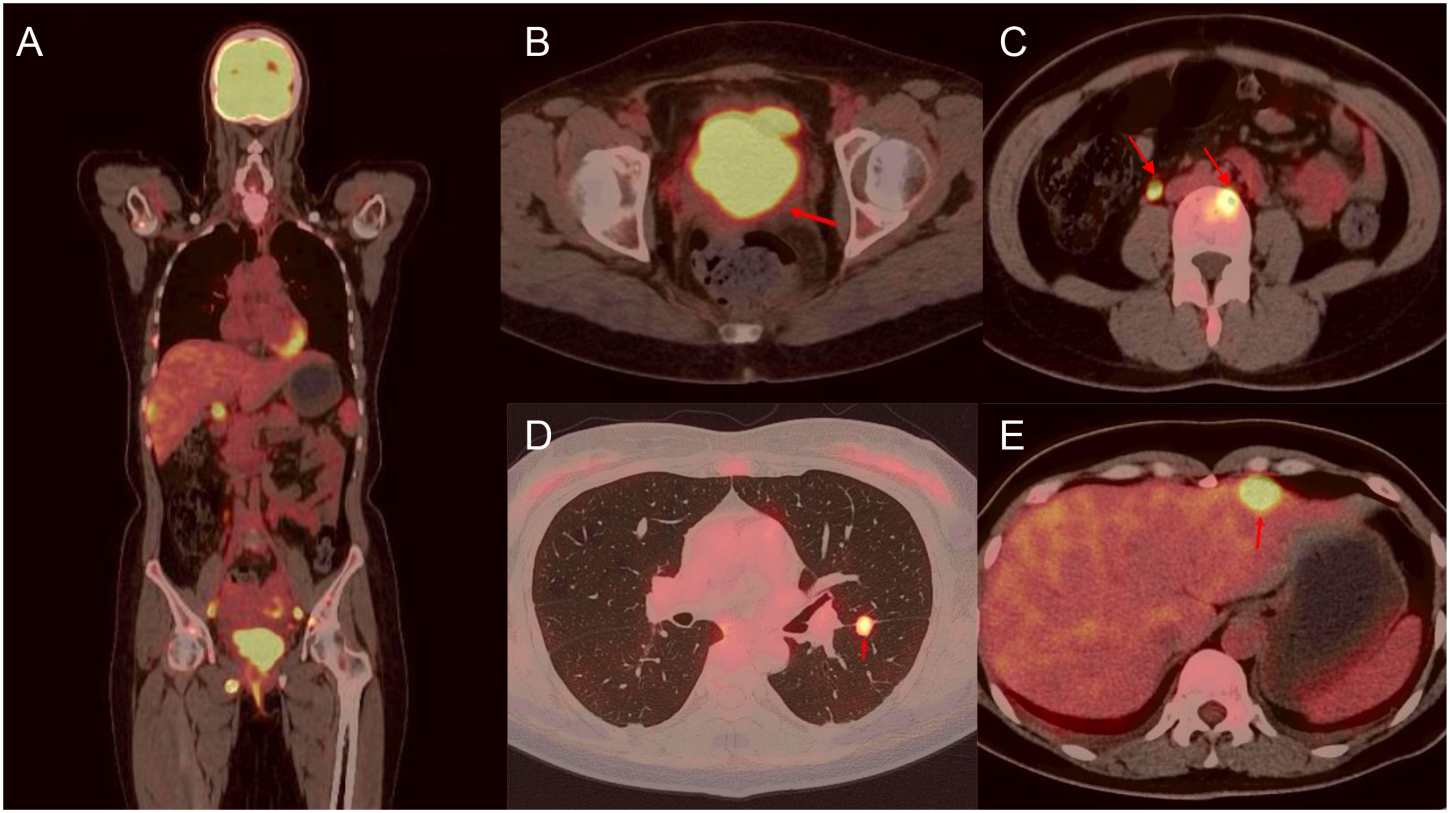
Fusion FDG-PET/CT cross section with a large lesion in the cervix and multiple metastatic lesions. There are representative images of lesions in the whole body **(A)**, cervix **(B)**, lumbar spine **(C)**, lung **(D)**, and liver **(E)**.

A 45-year-old woman presented in January 2024 with abnormal uterine bleeding (AUB). Gynecological examination revealed a tumor-like mass on the cervix, approximately 5 cm in diameter. The lesion extended to the vault and upper segment of vagina, with nodular thickening noted on the left parametrium. Outpatient doctor highly suspected a cervical tumor, and subsequent human papillomavirus (HPV) testing showed a high-risk HPV (hrHPV) 18-positive result. Further cervical biopsy and pathological examination indicated high-grade neuroendocrine carcinoma. Immunohistochemical (IHC) staining discovered positivity for p16, CD56, and Synaptophysin (Syn), with focal positivity for Chromogranin A (CgA). The Ki67 labeling index was approximately 90% (**Figure 1**). For tumor markers, both carcinoembryonic antigen (CEA, reference value<2.5 ng/mL) and neuron-specific enolase (NSE, reference value<16.3 ng/mL) levels were elevated, with values of 4.1 ng/mL and 76.1 ng/mL, respectively. Imaging studies showed extensive and advanced disease. Positron emission tomography/computed tomography (PET/CT) scans detected multiple metastases in the lung, liver, pelvic lymph nodes, and bones, including the humerus, scapula, sternum, ribs, thoracic spine, lumbar spine, ilium, ischium, pubis, and femur (**Figure 2**). At this point, the patient could be diagnosed with stage IVB NECC.

Due to the extensive primary tumor and multiple metastases, the patient was not a candidate for surgery. Palliative chemotherapy and radiotherapy were planned, with a therapeutic regimen consisting of cisplatin, paclitaxel, and bevacizumab, and the addition of tislelizumab for the PD-L1-positive tumor. The patient reported that previous physical examinations did not reveal any electrolyte disturbances. However, prior to the initiation of the first chemotherapy cycle, she was diagnosed with hyponatremia, with a serum sodium concentration of only 124.9 mmol/L. During chemotherapy administration, we provided adequate sodium supplementation and closely monitored the patient’s serum sodium levels, which dropped to a minimum of 114.8 mmol/L but slowly returned to normal. During the second chemotherapy cycle, she experienced symptoms of palpitations, chest tightness, hematemesis, and delirium. Laboratory findings indicated a serum sodium level of 112 mmol/L. After sodium replenishment, the patient’s symptoms improved, but the serum sodium level remained persistently low. Furthermore, despite subsequent rigorous monitoring and treatment, she spent very few days with normal sodium levels, and later developed pronounced limb weakness and finger numbness with stiffness. Additionally, the patient exhibited moderate hypocalcemia and mild hyperglycemia.

To investigate the cause of the refractory electrolyte imbalance, we conducted tests on the patient’s mineralocorticoids, glucocorticoids, plasma total cortisol (PTC), adrenocorticotropic hormone (ACTH), calcitonin, thyroid function indicators and sex hormones, all of which were within normal limits. But parathyroid hormone level rose to 102.7 pg/mL, urine sodium concentration increased to 237.3 mmol/L, and the plasma osmolality was 253.08 mOsm/L. Computed Tomography (CT) and Magnetic Resonance Imaging (MRI) results showed no structural changes in the hypothalamus, pituitary gland, and adrenal glands. Thyroid ultrasound disclosed a nodule approximately 1 cm in diameter, suggestive of nodular goiter with adenomatous nodules. To gain a deeper understanding of the patient’s genetic profile, NGS was performed on paraffin-embedded cervical tumor tissue and saliva to assess both germline and somatic variations. No pathogenic alteration was detected in the germline, but there were five clinically significant somatic variations, namely, PEAR1-NTRK1 fusion, decreased PTEN copy number, and increased MB21D2, FGF10, and RICTOR copy number (**Table 1**). The tumor was microsatellite instability-low (MSI-L) or microsatellite stable (MSS), and tumor mutational burden (TMB) was 1.13 Muts/Mb. The immunohistochemistry test results showed a Combined Positive Score (CPS) of 1, signifying PD-L1 positivity.

**Table 1:** Somatic variants of cervical tumor lesion detected by next-generation sequencing.

Gene	Result	Mutation abundance or copy number	Transcript	Clinical significance
PEAR1-NTRK1	Fusion PEAR1 (Promoter: IVS1)-NTRK1 (IVS10: end)	22.48%	NM_001080471.1- NM_002529.3	Yes
PTEN	decreased copy number	0.31	NM_000314.4	Yes
MB21D2	increased copy number	5.26	NM_178496.3	Yes
FGF10	increased copy number	4.92	NM_004465.1	Yes
RICTOR	increased copy number	3.88	NM_152756.3	Yes
NCOR1	p.S509L c.1526C>T	9.41%	NM_006311.4	Unknown
MAP3K4	p.G1366R c.4096G>A	7.14%	NM_005922.4	Unknown
ERBB4-PLEKHM3	Fusion ERBB4 (Promoter: EX25)-PLEKHM3 (IVS7: End)	1.12%	NM_005235.2- NM_001080475.2	Unknown
MGA	p.R2074H c.6221G>A	0.73%	NM_001164273.2	Unknown

**Figure 3:**
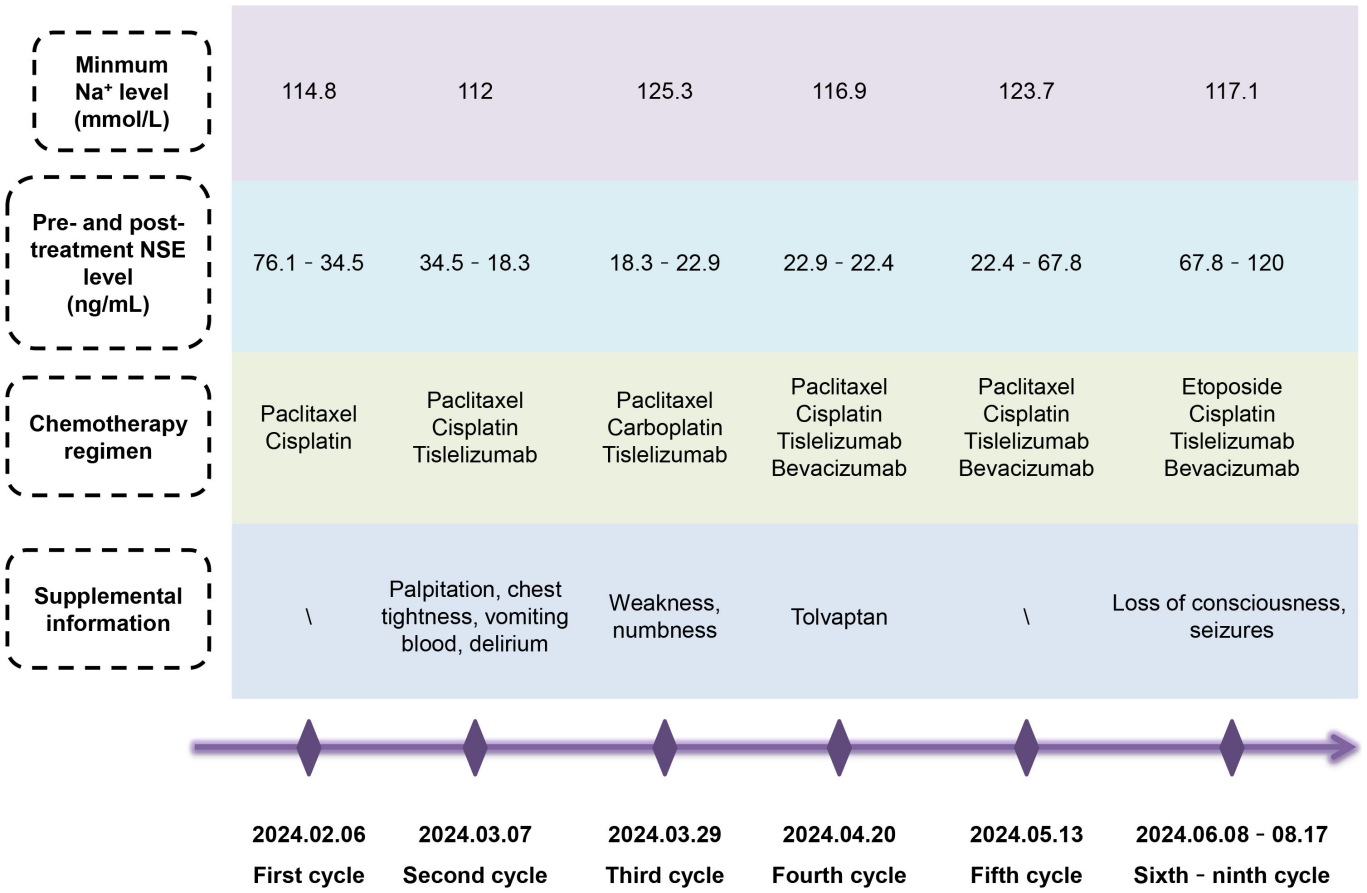
Summary of the patient’s condition over nine cycles of chemotherapy. Despite switching chemotherapy regimen, controlling fluid intake or rehydrating, and administering tolvaptan, the patient continued to experience persistent severe hyponatremia with grievous clinical symptoms, requiring long-term intravenous and oral sodium supplementation. After several fluctuations, the specific tumor marker NSE level ultimately increased.

Considering the potential nephrotoxicity of cisplatin and the stringent fluid intake requirements, which may precipitate hyponatremia, we replaced cisplatin with carboplatin during the third cycle of chemotherapy. Nonetheless, this change proved ineffective. Given the possibility of paraneoplastic syndrome SIADH presented in NENs, we curtailed fluid intake and empirically prescribed the V2R antagonist tolvaptan, but this did not prevent the continued loss of sodium. To date, the patient has undergone nine cycles of chemotherapy, targeted therapy, and immunotherapy (**Figure 3**). Moderate to severe hyponatremia persisted, requiring long-term intravenous and oral sodium supplementation. Unfortunately, the patient’s condition had progressed. During the first four cycles of chemotherapy (paclitaxel + cisplatin ± tislelizumab ± bevacizumab), the NSE level initially dropped significantly but then stabilized at a marginally elevated plateau of about 22 ng/mL. After the fifth cycle, the NSE level rose to 67.8 ng/mL. Consequently, from the sixth to the ninth chemotherapy cycles, we switched from the TP regimen (paclitaxel + cisplatin) to the EP regimen (etoposide + cisplatin), but NSE increased again to 120 ng/mL after two decreases. Although not specific, the CEA level continuously rose to 20.2 ng/mL. A follow-up CT scan three months after the initial treatment denoted a slight reduction in the cervical lesion, but new lesions appeared in multiple bones, including the clavicle, humerus, thoracic spine, and lumbar spine. The MRI scan seven months later suggested the possibility of brain metastasis. Due to disease progression, radiotherapy was not being considered.

## 3 Discussion

Refractory hyponatremia, accompanied by severe clinical symptoms and unresponsive to multiple treatments, is exceedingly rare in NECC. Firstly, the patient had developed hyponatremia prior to treatment, suggesting that hyponatremia may be one of the manifestations of paraneoplastic syndrome. Auxiliary examinations ruled out the possibility of primary or secondary adrenal insufficiency, hypothyroidism, and hypopituitarism. SIADH, caused by ectopic production of antidiuretic hormone (ADH) or by the enhanced effect of ADH due to anticancer drugs, leads to water retention, increased urinary sodium excretion, and dilutional hyponatremia. Given the patient’s decreased plasma osmolality, indicative of hypotonic hyponatremia, we administered treatments including fluid restriction and sodium supplementation. Studies demonstrated that the V2R antagonist tolvaptan was effective in treating SIADH in SCLC patients (10), so we adopted this approach as well. However, despite our efforts, the patient continued to experience ongoing sodium loss. Although there have been reports of secondary tolvaptan resistance in SIADH patients, which could signal a worsening condition (11), our patient showed no therapeutic response from the beginning. It raised concerns about other underlying mechanisms contributing to hyponatremia.

Secondly, chemotherapy drugs exacerbated the severity of sodium deficiency. In gynecological tumor chemotherapy, the most commonly used platinum drugs are cisplatin and carboplatin. Cisplatin, in particular, has stronger gastrointestinal and renal toxicity, both of which can potentially lead to sodium imbalance. A review of 137 esophageal cancer patients treated with cisplatin found that up to 59% developed hyponatremia (12). Similarly, in a retrospective study of 814 cisplatin-treated lung cancer patients, 83.7% experienced varying degrees of hyponatremia, with 7.7% having severe instances (13). K.R. Brown et al. reported a case of advanced large cell NECC with SIADH secondary to cisplatin, which resolved after switching to carboplatin (6). Therefore, in the third chemotherapy cycle, we opted for carboplatin, significantly reducing the patient’s gastrointestinal side effects, although notable hyponatremia persisted.

Furthermore, some rare and specific complications should be taken into consideration. RSWS, or renal salt wasting syndrome, is an infrequent side effect caused by platinum-based medications damaging the renal tubules. Similar to SIADH, RSWS involves the loss of sodium ions in the urine, but the differences are that RSWS is associated with extracellular volume depletion and often presents with hypokalemia, hypophosphatemia, and hypocalcemia. Serious cases may lead to hypotension. Management of RSWS typically includes replenishing blood volume and sodium salts, with the possibility of effectiveness of fludrocortisone (14). In an integrated analysis, the moderate hypocalcemia and transient hypokalemia observed in this patient were likely of minimal clinical significance, and her normovolemic status was also inconsistent with RSWS. Patients with cerebral salt wasting syndrome (CSWS) generally exhibit reduced blood volume, often resulting from neurological injury, tumors or surgery (15). But, in this case, a cranial CT scan was performed and no abnormalities were found.

Additionally, we were attempting to identify the potential genetic causes of hyponatremia in this patient. Through NGS, PEAR1-NTRK1 fusion and copy number changes in PTEN, MB21D2, FGF10, and RICTOR were detected. Frumovitz M et al. sequenced tumor-associated genes in 44 patients with high-grade NECC and found that PIK3CA (alterations in 18% of patients), KRAS (14%), and TP53 (11%) were the most commonly mutated genes (16). The somatic variants ascertained in this case were relatively infrequent in NECC, with mutations in PTEN, FGF10 and RICTOR previously reported (17, 18). Rapamycin-insensitive companion of mTOR (RICTOR) is a major subunit that makes up mTOR2, and the role of RICTOR/mTORC2 in regulating cell growth, metabolism, and ion transport by responding to growth factors, cytokines, and hormones has been extensively studied. It can influence the epithelial Na+ channel in the alveolar epithelium, distal nephron and endometrium to control Na+ transport (19–21). Whether increased RICTOR copy number in this patient contributed to disrupting Na+ balance required further research and consideration. Regrettably, a review of the literature did not reveal any correlation between other molecular changes and electrolyte disturbances.

In summary, addressing NECC patients with intractable hyponatremia of unknown etiology and poor efficacy is exceedingly challenging. We believe that routine electrolyte monitoring is crucial for NECC patients, as early detection of electrolyte imbalances can significantly impact treatment outcomes. When necessary, consultation with specialists, such as endocrinologists or Intensive Care Unit (ICU) physicians, should be sought to assist in both diagnosis and management. Actively identifying the underlying cause is critical, as different diseases require distinct or even opposite treatments, such as SIADH and RSWS. Sequencing techniques could be employed to explore the mechanisms at the genetic level. From a treatment perspective, if tumor lesions could be surgically removed, patients might temporarily be relieved from hyponatremia. However, in this case, surgery was not an option due to the advanced stage and extensive metastasis. For these patients, avoiding medications that disrupt sodium balance is critical, and close monitoring of electrolytes should be maintained both in and outside the hospital. Long-term treatment planning and comprehensive management are also necessary. All of these measures aim at improving the patient’s quality of life and prognosis.

## Data Availability

The original contributions presented in the study are included in the article/supplementary material. Further inquiries can be directed to the corresponding author.
